# Complete Genome Sequence and Methylome Analysis of Thermoactinomyces vulgaris 2H

**DOI:** 10.1128/MRA.00657-19

**Published:** 2019-08-08

**Authors:** Houda Mankai, Brian P. Anton, Victoria Wu, Tamas Vincze, Richard J. Roberts, Ferid Limam, Alexey Fomenkov

**Affiliations:** a Laboratoire des Substances Bioactives, Centre de Biotechnologies de Borj Cédria, Hammam Lif, Tunisia; b New England Biolabs, Inc., Ipswich, Massachusetts, USA; c Wellesley College, Wellesley, Massachusetts, USA; University of Maryland School of Medicine

## Abstract

Here, we report the complete genome sequence and full methylome analysis of a newly isolated, aerobic, thermophilic, Gram-positive actinomycete, a strain of Thermoactinomyces vulgaris designated strain 2H.

## ANNOUNCEMENT

An aerobic, Gram-positive, thermophilic actinomycete, a strain of Thermoactinomyces vulgaris designated strain 2H, was isolated from a soil sample collected from the southwestern part of Tunisia (Tamaghza, Tozeur). The strain isolation was carried out using a dilution agar plating method on tryptic soy agar (TSA) medium (pH 7.3 ± 0.2). Ten grams of soil sample was suspended in 100 ml sterile distilled water and mixed. Further 10-fold dilutions were prepared, and a 0.1-ml sample from each dilution was spread onto the TSA plates with slight modifications (1). After incubation at 55°C for 5 days, a *Thermoactinomyces*-like strain was isolated and purified by streaking on new plates on the same medium; it was stored at 4°C.

The 16S rRNA gene sequence analysis of strain 2H (GenBank accession number KJ817372) using the EzBioCloud database (2) showed that the organism was closely related to Thermoactinomyces vulgaris DSM 43016^T^ (AF138739) (97.90% similarity) and Thermoactinomyces intermedius DSM 43846^T^ (AF138734) (97.41%). DNA-DNA hybridization confirmed that strain 2H belongs to the species *T. vulgaris*, and we have designated it *T. vulgaris* 2H. Strains with this designation are a potential source of thermostable enzymes such as proteases (3).

Genomic DNA from a culture of *T. vulgaris* 2H in tryptic soy broth (pH 7.3 ± 0.2), in shaken flasks (120 rpm) at 55°C, was purified with a GF-1 bacterial DNA extraction kit (Vivantis Technologies Sdn. Bhd., Malaysia) and sequenced using the Pacific Biosciences (PacBio) RS II sequencing platform. Briefly, SMRTbell libraries were constructed from a genomic DNA sample sheared to ∼20 kb using the g-TUBE protocol (Covaris, Woburn, MA, USA), end repaired, and ligated to PacBio hairpin adapters. Incompletely formed SMRTbell templates and linear DNAs were digested with a combination of exonuclease III and exonuclease VII (New England Biolabs, Ipswich, MA, USA). DNA qualification and quantification were performed using a Qubit fluorometer (Invitrogen, Eugene, OR, USA) and a 2100 Bioanalyzer (Agilent Technologies, Santa Clara, CA, USA). One 12-kb SMRTbell library was prepared according to modified PacBio sample preparation protocols, including additional separation on a BluePippin instrument (Sage Science, Beverly, MA, USA), and sequenced with C4-P6 chemistry. Two single-molecule real-time (SMRT) cells were sequenced, one with a non-size-selected (12-kb) library and one with a size-selected 14-kb library, with a 300-minute collection time. The sequencing reads (123,762), with a mean subread length of 8,746 bp and yield of 1.082 Gb, were *de novo* assembled using the HGAP_Assembly.3 version 2.3.0 with default quality and read length parameters and polished using Quiver (4). The polished assembly generated 2 closed-circular genome elements with 48% GC content for the main chromosome (2,619,280 bp) and 41.9% GC content for the plasmid (18,747 bp). The assembled sequence was annotated using the NCBI Prokaryotic Genome Annotation Pipeline (PGAP) (5, 6).

One advantage of the SMRT sequencing platform is its ability to detect the epigenetic state of sequenced DNA (7–9). Three apparent DNA methyltransferase recognition motifs, CCTGGVDCR, GGCCANB, and GGCCBBVNY, were detected by SMRT motif and modification analysis version 2.3.0, and each was reported to contain m4C modifications. However, this is a common artifact of PacBio motif calling; in fact, these motifs must originate from the genuine motifs CCWGG and GGCC as determined separately by genomewide m5C methylation analysis (B. P. Anton, unpublished data). Additional scanning with the SeqWare program (10) showed that the strain does not bear any m6A or m4C methyltransferase genes that might otherwise have been responsible for these motifs. All the m5C motifs were matched with the responsible methyltransferases, and the results are shown in Table 1 and have been deposited in REBASE (11).

**TABLE 1:** Summary of genome elements, methyltransferase genes, and motifs identified in Thermoactinomyces vulgaris 2H^*a*^

Genetic element of Thermoactinomyces vulgaris 2H	Accession no.	Genome size (bp)	Genome coverage (×)	Methylase (RM system) name	Recognition motif^*b*^	Methylation, RM type
Chromosome	CP039710	2,619,280	328.15	RM.Tvu2HI RM.Tvu2HII M.Tvu2HOR8305P M.Tvu2HORF1205P	GG**C**C C**C**WGG Inactive system Frameshift	5mC, II 5mC, II 6mA, I 5mC, II
Plasmid pTvu19	CP039711	18,747	606.42	None		

a RM, restriction-modification.

b Modified bases are shown in bold, or the base opposite to them is underlined.

### Data availability.

The complete genome sequence of Thermoactinomyces vulgaris 2H is available in GenBank under the accession numbers CP039710 and CP039711. The original sequence reads have been deposited at NCBI under the SRA accession numbers SRR8979896 and SRR8979897. The BioProject accession number is PRJNA534300.
